# Oral Symptoms and Oral Health-Related Quality of Life in People with Rare Diseases in Germany: A Cross-Sectional Study

**DOI:** 10.3390/ijerph15071493

**Published:** 2018-07-15

**Authors:** Sabrina Wiemann, Nicolas Frenzel Baudisch, Rainer A. Jordan, Johannes Kleinheinz, Marcel Hanisch

**Affiliations:** 1 Department of Cranio-Maxillofacial Surgery, Research Unit Rare Diseases with Orofacial Manifestations (RDOM), University Hospital Münster, Albert-Schweitzer-Campus 1, Building W 30, D-48149 Münster, Germany; s_wiem07@uni-muenster.de (S.W.); johannes.kleinheinz@ukmuenster.de (J.K.); 2 Institute of German Dentists (IDZ), Research Focus: Sociology of medicine and Health Psychology, Universitätsstraße 73, D-50931 Köln, Germany; n.frenzel@idz.institute (N.F.B.); r.jordan@idz.institute (R.A.J.)

**Keywords:** Rare diseases, quality of life, oral health, OHIP-14, OHRQoL, patient reported, outcome

## Abstract

*Background*: The aim of this study was to collect information on oral health-related quality of life (OHRQoL) in people with rare diseases. *Methods*: A questionnaire comprising free text questions and the German version of the standardized Oral Health Impact Profile-14 (OHIP-14) questionnaire on OHRQoL was developed. All participants who indicated oral symptoms in the questionnaire were included in a cluster analysis. Different cluster analyses were performed (Ward’s, k-Means) to find symptom profile groups in the data. *Results*: A total of 484 questionnaires with 96 rare diseases were included in the study. The most reported symptoms were anomalies of the tooth formation, dysgnathia, changes in number of the teeth, and malocclusions. The OHIP mean values of the five resulting symptom clusters ranged from 15.1 to 19.9, which is very high compared to the general population in Germany, which has a mean value of 4.09. *Discussion*: All investigated symptoms show a negative association with OHRQoL, but the strongest were for symptoms of the oral mucosa and periodontal diseases. All the symptoms described in this cluster analysis can lead to considerably higher mean values of the OHIP total score among people with rare disease and thus to worse OHRQoL than reported in the general population.

## 1. Introduction

Rare diseases are by definition ‘rare’. A disease is categorized as ‘rare’ if fewer than 2000 people suffer from it. In the European Union, between 27 and 36 million people have a rare disease [1,2], about four million of which live in Germany [3].

Studies have shown that about 15% of all known 6000–8000 rare diseases can manifest orofacially [4,5,6] and can affect oral health-related quality of life (OHRQoL) [7]. In 2009, the Council of the European Union issued a recommendation to member states to develop measures to improve medical care for people with rare diseases [8]. As a result, the National Action Plan for People with Rare Diseases, which is currently in the implementation phase, was launched in Germany in 2013 [9].

Thus far, there have been very few studies on the OHRQoL of people with rare diseases; the few previous analyses reports showed reduced OHRQoL in people with rare disease [7,10,11].

The Oral Health Impact Profile (OHIP) is a validated instrument to assess OHRQoL; with 14 items, the OHIP-14 measures the incidence of different functional and psychosocial influences on OHRQoL. The maximum number of points is 56 in the OHIP-14 version, which means a very high impact on OHRQoL for the patient [12]. Therefore, this serves as a suitable instrument for describing the influence of oral health on general health from the patient’s perspective [13].

On average, it takes seven years before a rare disease is correctly diagnosed [14]. In the process, oral manifestations of rare diseases seem to negatively influence OHRQoL [7]. With oral symptoms, the dentist can contribute towards reducing the time taken for a diagnosis. Dental checkups make up one of the most frequent reasons for contact between patients and health care professionals. In cases of oral symptoms, the dentist can be the first health care professional who is able to identify a rare disease by its oral manifestations [15]. This could in turn positively influence OHRQoL and also general quality of life, because dentists and physicians can start the appropriate therapy earlier. There is a low-frequency problem in the context of individual rare diseases. Due to that low frequency, analyses with a high number of cases for individual diseases are difficult, which is why a cluster analysis based on symptoms was considered appropriate for this study. With this method it is possible to investigate all diseases with oral manifestations in an overarching study and not every disease on its own. Symptom profile groups allow us to find out which symptoms have a negative impact on OHRQoL. A cluster analysis allows the possibility of finding symptom profile groups in the present data source.

Therefore, the aim of this study was to collect information on OHRQoL in people with rare diseases and to find out which symptoms were mostly reported and which symptoms had the highest impact on OHRQoL.

## 2. Methods

The present study was conceived as an epidemiological survey to collect information on OHRQoL in people with rare diseases in Germany. A questionnaire comprising free text questions and the German version of the standardized OHIP-14 questionnaire on OHRQoL was developed [16]. The free text questions were chosen so that the participants could provide their information without prescribed response options from the principal investigator. The questionnaire (Table S1) was sent digitally to all 125 member associations of the umbrella organization of self-help groups, the Alliance of Chronic Rare Diseases (ACHSE e.V.) in Germany in February 2016. All questionnaires received by July 2017 were considered. Approval obtained from the Ethics Commission of the General Medical Council of Westphalia-Lippe and the Westphalian Wilhelms University of Münster is available (Ref. No. 2106-006-f-S). Included in the study were all people in Germany with a rare disease aged 16 and above who could be reached via the ACHSE.

### 2.1. Questionnaire

The OHIP-14 questionnaire is the short form of the OHIP-49 and gathers information on the frequency of functional limitations, pain, psychological unease/discomfort, physical impairment, mental impairment, social impairment, and disadvantage/disability in the past month [16]. Each of the 14 items was coded as follows: 0 = never, 1 = hardly ever, 2 = now and then, 3 = often, and 4 = very often. In the open-ended questions, possible oral manifestations of the disease were also requested. To streamline analysis, the principal investigators translated the patient responses into medical terms and divided them into categories. For instance, the subjective patient statement ‘dry mouth’ was renamed xerostomia/hyposalivation.

### 2.2. Participants

There was no patient selection; all self-help groups listed with the ACHSE were contacted and all persons with a rare disease who are in contact with a self-help organization listed with the ACHSE could participate in the study. As a result, individual rare diseases, depending on the number of participants, are represented more strongly or less strongly in this study. The aim was to get as many questionnaires as possible during the study period. Due to the rarity of individual rare diseases, even rare diseases for which only one questionnaire was submitted were included in the study. Against this backdrop, the attempt was to focus on the symptoms and their effect on OHRQoL in order to also consider the dependency of the OHRQoL on the symptom constellation.

### 2.3. Data Source

All participants who indicated oral symptoms in the questionnaire were included in the cluster analysis. Participants who did not indicate oral symptoms were excluded (46.5%). The cluster analysis was executed using the following 12 symptoms, which were translated into medical terminology based on the data of the participants: dysgnathia, malocclusion, anomalies of tooth formation, mineralization disorders of the teeth, cleft lip or cleft palate, microstomia, temporomandibular dysfunction, diseases of the oral mucosa, diseases of the periodontium, changes in the number of teeth, neurological diseases, and vegetative disorders. These were coded as dichotomous variables suitable for a cluster analysis both in their incidence and in their medical relevance.

### 2.4. Statistical Methods

The statistics program SPSS was used for the analysis. The aim of a cluster analysis is that it forms groups of cases according to their similarities and dissimilarities in certain aspects. In the present study, these aspects were the participants’ oral symptoms so that the mathematical algorithms yielded symptom profile groups. Each group is very different from the others but is itself highly homogeneous [17].

First, a cluster analysis using the Ward algorithm was used with an indefinite number of clusters. With the Elbow criterion, 2–6 clusters seemed to be an adequate number of clusters. Based on this criterion, a cluster analysis applying the k-means algorithm with 2–6 clusters and another Ward’s analysis with 2–6 clusters were then carried out. Both analyzing methods were compared with regard to group belonging, group size, and the symptom groups in the individual clusters. The Kappa coefficient (4, 04) was also calculated. This coefficient shows the consensus of the cluster solutions [17]. Due to the data’s heterogeneity, the results were considered acceptable.

Several cluster analyses were performed with cases ordered randomly, and all analyses showed stable results independent of the order of cases and algorithm (Ward’s, k-Means) used. After comparing the symptom prevalence in each cluster of every cluster analysis solution and according to medical and/ or dental considerations, Ward’s method with five clusters was chosen for further analysis. For instance, dysgnathia and malocclusion both appear with high prevalence in cluster five; from a medical perspective, there is a logical connection between these two symptoms; therefore, this resulting cluster was deemed appropriate. Thus, the five-cluster Ward’s method revealed the most interpretable and consistent clusters. The five resulting clusters of the k-Means cluster solution showed nearly the same symptom profile groups with only slightly less marked symptom prevalences. Following this, for each cluster, the mean, median, 95% confidence interval, standard deviation, and minimum and maximum of the OHIP total score was calculated. We performed a cluster analysis on group symptoms and formed symptom profile groups to solve the low-frequency problem of rare diseases and to get an overview of which symptoms co-occur with each other.

## 3. Results

Of the 545 submitted questionnaires, 484 could be included in the study; 61 questionnaires could not be considered because the participants were less than 16 years old. Twelve self-help groups refused to participate in the study either due to lack of interest or because the self-help group believed that the study would be of no personal benefit to the members. The participants were predominantly female (64.7%), and their ages ranged from 16 to 80 years with an average of 44.6 years. The OHIP mean values of the resulting symptom clusters ranged from 15.1 to 19.9. In total, participants with 96 different rare diseases participated in the study.

All rare diseases analyzed in this study are listed in the Orphanet classification of rare diseases [18], and the most prevalent (n ≥ 10) are listed in Table 1. The diseases were recorded based on data given by the patients. The data of 257 participants who described an oral manifestation were analyzed by cluster analysis. Of the 95 named symptoms, which were indicated by the patients in their own words, the 12 symptoms that were represented most frequently and could have an impact on OHRQoL from a medical perspective were included in the cluster analysis (Table 2).

Cluster 1 included anomalies of tooth formation (conical shaped teeth, taurodontism, macrodontia, and microdontia) and mineralization disorders of the dental hard tissue (amelogenesis imperfecta, dentine dysplasia, and dentinogenesis imperfecta). The mean value of the OHIP total value was 15.9 (12.6; 19.2) points.

Cluster 2 included symptoms involving the oral mucosa (aphthae and stomatitis) and diseases of the periodontium (gingivitis, periodontitis, recessions, hyperplasia of the oral mucosa, tooth loss through periodontitis) and microstomia. The mean value of the OHIP total value was 19.7 (13.8; 25.7) points. It seems that symptoms related to the oral mucosa and diseases of the periodontium have the highest impact on OHRQoL.

Cluster 3 is characterized by the symptom changes in the number of teeth (hypodontia, oligodontia, anodontia, and hyperdontia), anomalies of tooth formation (form abnormalities, macrodontia, microdontia, and taurodontism), and cleft lip and palate. The mean value of the OHIP total value was 16.9 (13.0; 20.8) points.

Cluster 4 included temporomandibular dysfunctions (bruxism, luxations, cartilage displacement, ankylosis, and pain in the mandibular joint), vegetative disorders (hyposalivation, hypersalivation, and xerostomia), and neurological diseases (facial nerve paresis, trigeminal neuralgia, and somatosensory disorders of the trigeminal nerve). The mean value of the OHIP total value was 16.9 (13.0; 20.8) points.

Cluster 5 included dysgnathia (micrognathia, macrognathia, high palate, and mandibular retrognathia) and malocclusions (crossbite, edge-to-edge bite, overbite, impacted tooth, inclination, wide space, and overcrowding). The mean value of the OHIP total value was 15.1 (11.6; 18.8) points.

**Figure 1:**
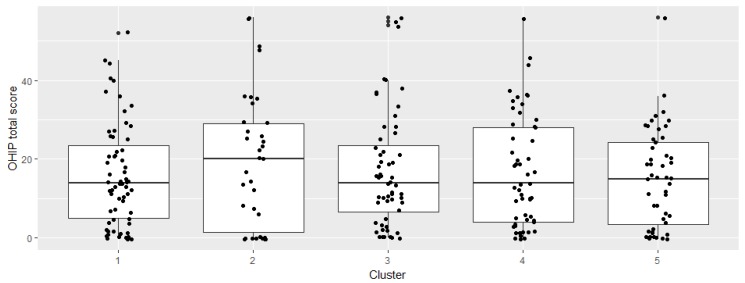
Boxplots of OHIP total score in each cluster.

If symptoms appear involving the oral mucosa, the periodontium, and a restricted mouth opening (microstomia), as is the case in cluster 2, this results in the worst OHIP total scores. The OHIP mean values in the other clusters vary only marginally (Table 3). Figure 1 shows the cluster distribution. 

## 4. Discussion

Not only can rare diseases negatively influence the general quality of life, they also negatively impact the OHRQoL [20]. The OHIP mean values in the present study among people with rare diseases had a mean value between 15.1 and 19.9 depending on the different symptom combinations. With cluster analysis techniques, we identified five symptom profile groups to analyze. The aim of this study was to group symptoms across all mentioned diseases and investigate the impact on OHRQoL.

### 4.1. Limitations

Because there are up to 8000 different rare diseases globally [21], it is impossible to generalize ‘rare diseases’ as ‘a’ disease, because each disease is peculiar, and furthermore, there are diverse manifestations within a rare disease [11,22].

Because the questionnaire was distributed using the snowball method, we cannot accurately say how many people were reached; however, we opted for this method due to the difficulty of reaching many people with rare diseases.

In addition, it is not possible to give evidence about the severity of the disease because the questionnaire only asks which diseases and symptoms the participants have. In a cluster analysis, it is difficult to group a large number of symptoms accordingly. The more symptoms included in a cluster analysis, the more difficult and unstable the cluster analysis becomes. Therefore, it is more insightful to limit the number of symptoms included in the analysis to enable a sensible formation of groups. This requires that symptoms that could also be significant to OHRQoL were not taken into consideration. In addition, a cluster analysis only groups symptoms and does not assess causal relationships. For this reason, no such assertions were made with respect to symptoms and OHRQoL [17].

This study therefore serves as an attempt to obtain a classification of symptoms across illnesses. Certain symptom combinations were found in the cluster analysis that are often mentioned together or possibly trigger each other. Coherence within a cluster was considered appropriate, because, for instance, microstomia complicates oral hygiene and hence promotes a disease of the periodontium. The mere presence of a symptom contains no information about its severity and intensity and therefore its effect on the OHRQoL.

### 4.2. Interpretation

In a representative study of the whole German population, an OHIP value of 4.09 points was established [19]. The participants of our study show a considerably worse OHRQoL than the general population.

If one considers the confidence intervals of the total German population, there is a significant difference from the people with rare diseases in our study. The confidence interval among patients with implanted dentures was between 10 and 12 points in the 90%. In our study, the confidence intervals only start at 12.6 points in clusters 1–4. Therefore, there are no overlaps of the intervals, which means significantly worse OHIP scores among people with rare diseases and oral symptoms.

The confidence intervals very slightly overlap in the lower boundary in cluster 5. The values tend to approach the 90th percentile of patients with removable dentures. The confidence interval there is between 15 and 19 points. It should be noted here that these are the worst 10% values in the total population, while these only correspond to the average values among people with rare diseases. However, maximum values of 56 points were also indicated in the current sample, which shows how strongly people with rare diseases can be impaired in their OHRQoL.

Our values also display strong scattering. For instance, the standard deviation in cluster 2 is 17 points. This means people in this cluster differ from the mean of 20 OHIP points by 17 points on average, which means 3–37 points. The participants with 3 points report a somewhat low impact on OHRQoL, while participants with 37 points report a high impact on OHRQoL [19].

The average OHIP total score was an average of between 15.1 and 19.9 of 56 possible OHIP points across all clusters. It is useful to understand what 1 OHIP point stands for. Reissmann et al. [23] have investigated how many events are necessary for 1 more OHIP point to be added by participants. The OHIP-49 questionnaire was used for their study, which does not allow for a direct comparison to the present study, but it does give an impression of the considerably reduced OHRQoL. They concluded that a total of 15.2 events are required in a month on average for the OHIP total score to increase by one point. Therefore, a person must interrupt a meal, feel unwell, or have pain an average of 15.2 times per month for the OHIP score to rise by 1 point. This means that the person must struggle with this constraint every two days.

Clearly, all symptoms are accompanied by reduced OHRQoL. The method of cluster analysis was chosen so that, without the influence of the principal investigators, it could be discovered which symptom combinations were indicated together in order to then describe varying OHRQoL between the groups.

Cluster analysis seems to be a suitable option for finding symptomatically homogeneous groups in larger samples regarding rare diseases [17]. In all the formed clusters, the OHIP total score has a mean value between 15.1 and 16.9 points; only in cluster 2 (diseases of the oral mucosa, diseases of the periodontium, and microstomia) is the mean value considerably above that at 19.9 and thus describes worse OHRQoL. This can be explained by the fact that diseases of the periodontium and diseases involving the oral mucosa, such as recurrent aphtous stomatitis [24] or epidermolysis bullosa, which is also accompanied by a restricted mouth opening, lead to pain and functional limitations and thus negatively influence the OHRQoL [25]. A microstomia, as can appear in scleroderma for instance, complicates oral hygiene in turn and thus favors the occurrence of periodontal diseases. A small mouth opening can also complicate or limit food intake and even make dental treatment problematic [25]. It is therefore plausible that rare diseases involving the oral mucosa, the periodontium, and a limited mouth opening may be accompanied by reduced OHRQoL.

The other clusters vary only marginally in the OHIP mean values. The symptom anomalies of tooth formation and mineralization disorders of the dental hard tissue grouped in cluster 1 resulted in an average OHIP total score of 15.9 (Table 3). Anomalies of tooth formation, such as microdontia, macrodontia, conical teeth, and taurodontism, lead to aesthetic restrictions, which negatively impact social life [26] and may require dento-aesthetic rehabilitation [27]. Mineralization disorders of the dental hard tissue, such as amelogenesis imperfecta and dentinogenesis imperfecta, not only lead to higher susceptibility to caries but may also cause pain and aesthetic limitations and negatively impact the confidence of the patients thus resulting in reduced OHRQoL [28].

The mean value of 16.9 was seen in cluster 3, where anomalies of tooth formation appear in conjunction with changes in the number of teeth and cleft lip and palate. The combination of multiple dental agenesis, anomalies of tooth formation, and cleft lip and palate has often been reported [29] and occurs generically in the Ectrodactyly–Ectodermal Dysplasia–Cleft Lip/Palate (EEC) syndrome [30]. Patients with congenital dental agenesis thus benefit with regard to their OHRQoL from dento-prosthetic rehabilitation, especially if the dentures are anchored to dental implants [31]. It is well documented that improvement of OHRQoL can be achieved through dental implants [32,33]. By contrast, if there has been no dento-prosthetic treatment for multiple dental agenesis, OHRQoL is considerably worse [34]. Cleft lip and palate lead to aesthetic and functional impairments, especially in food intake and articulation and negatively affect OHRQoL [35]. It therefore seems plausible that, among people with anomalies of tooth formation, dental agenesis, and cleft lip and palate, not only aesthetic and psychosocial impairments but also functional limitations can negatively influence OHRQoL.

The symptoms of temporomandibular dysfunction, vegetative disorders, and neurological diseases are presented in cluster 4. Shooting pain several times daily, as occurs in trigeminal neuralgia, negatively impacts OHRQoL just like temporomandibular dysfunctions, with the resulting pain and functional limitations [36,37]. A reduced rate of saliva flow (hyposalivation) or the absence of saliva flow (xerostomia) also has a negative effect on OHRQoL [38]. Rare diseases such as Sjögren syndrome, which are accompanied by a reduced rate of saliva flow, also show high rates of caries, which are caused wholly or partly by reduced saliva flow [39]. The OHIP total score was an average of 16.9 in cluster 4. Due to the considerable severity of some of the symptoms (shooting pain in the area surrounding the trigeminal nerve in trigeminal neuralgia, as well as food intake complicated by mouth dryness and the often-existing mucositis), it would have been expected that particularly high OHIP values would be seen in this cluster. However, the mean value of 16.9 is only slightly higher than that in clusters 1, 3, and 5 (15.9, 16.9, 15.1) and is lower than the values of cluster 2. A possible reason for this is data heterogeneity. Another possible reason is symptom specificity. There was no question about the severity of the symptom, which is a subjective statement from the participants.

Dysgnathia, in combination with malocclusions, displayed the lowest mean value of the OHIP total score at 15.19, as seen in cluster 5. Therefore, in this sample, dysgnathia and malocclusions affect OHRQoL less negatively than symptoms in other clusters. A possible explanation could be that only slight forms of dysgnathia or malocclusion were present among the participants of the study, because the negative influence on OHRQoL increases with the severity of the malocclusion [40].

In general, worse oral health of people with rare diseases could be reported in this study, as it is known in single rare diseases such as lysosomal storage disease [41].

In the future, more nuanced surveys, with higher numbers of cases for individual rare diseases and estimates for the severity of the symptoms, are still necessary in order to evaluate the OHRQoL more specifically.

## 5. Conclusions

All investigated symptoms are associated with relatively low OHRQoL. The symptoms related to diseases of the periodontium, diseases of the oral mucosa, and microstomia are associated with lower OHRQoL in people with rare diseases. Cluster analysis is a suitable method for creating symptom groups. All symptoms described in this cluster analysis may lead to considerably higher mean values of the OHIP total score among people with rare diseases and thus to worse OHRQoL than are described in the average population.

## Figures and Tables

**Table 1:** Commonly presented diseases in the present study (n ≥10).

Name of Disease	OMIM Number	Number of Individuals
Marfan Syndrome	154700	51
Ectodermal Dysplasia	305100	46
Achalasia	231550	44
Sarcoidosis	181000	36
Ehlers–Danlos Syndrome	120180	32
Lymphangioleiomyomatosis	606690	17
Alpha-1-Antitrypsin Deficiency	613490	15
Syringomyelia	118420	13
X-linked Hypophosphatemia	307800	13

**Table 2:** Prevalences of the analyzed symptoms in the sample (%).

Symptoms	Prevalence (%)
Anomalies of the Tooth Formation	24.4
Dysgnathia	18.4
Changes in Number of the Teeth	13.6
Malocclusion	12.4
Oral Mucosa Disease	9.5
Mineralization Disorders of the Hard Tissue of the Teeth	9.3
Temperomandibular Dysfunction	5.4
Disease of the Periodontium	5.4
Vegetative Disorders	3.1
Neurological Disease	2.1
Cleft Lip and Palate	1.7
Microstomia	1.2

**Table 3:** Categorization of the five clusters with symptom prevalence in each cluster in % and Oral Health Impact Profile-14 (OHIP-14) mean, standard deviation, minimum, and maximum. For reference: mean OHIP total score of the general German population is 4.09 [19].

Clusters	Symptom Prevalence in Each Cluster (%)	Number of Individuals in Each Cluster	OHIP Mean (Minimum; Maximum)	OHIP Standard Deviation
Cluster 1	Anomalies of the Tooth Formation: 98% Mineralization Disorders of the Hard Tissue of the Teeth: 70%	63	15.9 (0;52)	13.1
Cluster 2	Oral Mucosa Disease: 83% Disease of the Periodontium: 34% Microstomia: 14%	35	19.7 (0;56)	17.1
Cluster 3	Changes in the Number of the Teeth: 100% Anomalies of the Tooth Formation: 96% Cleft Lip/Palate: 7%	56	16.9 (0;56)	14.5
Cluster 4	Temporomandibular Dysfunctions: 31% Vegetative Disorders: 16% Neurological Diseases: 15%	55	16.9 (0;56)	14.3
Cluster 5	Dysgnathia: 100% Malocclusion: 54%	48	15.1 (0;56)	12.4

## Supplementary Materials

The following are available online at http://www.mdpi.com/1660-4601/15/7/1493/s1, **Table S1**: Original version of the questionnaire.
