# Supplementary material for: Oral Symptoms and Oral Health-Related Quality of Life in People with Rare Diseases in Germany: A Cross-Sectional Study
Source: Int J Environ Res Public Health. 2018 Jul 15;15(7):1493. doi: 10.3390/ijerph15071493 (PMC6068528; doi:10.3390/ijerph15071493)
Supplement: Supplementary file 1 [file ijerph-15-01493-s001.docx]

**Supplement file:**

| **Hatten Sie im vergangenen Monat aufgrund von Problemen**  **mit Ihren Zähnen, im Mundbereich oder mit Ihrem Zahnersatz …** | **sehr**  **oft** | **oft** | **ab**  **und zu** | **kaum** | **nie** |
| --- | --- | --- | --- | --- | --- |
| ….Schwierigkeiten bestimmte Worte auszusprechen? |  |  |  |  |  |
| ….das Gefühl, Ihr Geschmackssinn war beeinträchtigt? |  |  |  |  |  |
| ….den Eindruck, dass Ihr Leben ganz allgemein weniger zufriedenstellend war? |  |  |  |  |  |
| ….Schwierigkeiten zu entspannen? |  |  |  |  |  |
| **Ist es im vergangenen Monat aufgrund von Problemen mit Ihren Zähnen, im Mundbereich oder mit Ihrem Zahnersatz vorgekommen, …** | **sehr**  **oft** | **oft** | **ab**  **und zu** | **kaum** | **nie** |
| ….dass Sie sich angespannt gefühlt haben? |  |  |  |  |  |
| ….dass Sie Ihre Mahlzeiten unterbrechen mussten? |  |  |  |  |  |
| ….dass es Ihnen unangenehm war, bestimmte Nahrungsmittel zu essen? |  |  |  |  |  |
| ….dass Sie anderen Menschen gegenüber eher reizbar gewesen sind? |  |  |  |  |  |
| ….dass es Ihnen schwergefallen ist, Ihren alltäglichen  Beschäftigungen nachzugehen? |  |  |  |  |  |
| ….dass Sie vollkommen unfähig waren, etwas zu tun? |  |  |  |  |  |
| ….dass Sie sich ein wenig verlegen gefühlt haben? |  |  |  |  |  |
| ….dass Ihre Ernährung unbefriedigend gewesen ist? |  |  |  |  |  |
| **Hatten Sie im vergangenen Monat …** | **sehr**  **oft** | **oft** | **ab**  **und zu** | **kaum** | **nie** |
| ….Schmerzen im Mundbereich? |  |  |  |  |  |
| ….ein Gefühl der Unsicherheit in Zusammenhang mit Ihren Zähnen, Ihrem Mund oder Ihrem Zahnersatz? |  |  |  |  |  |

OHIP-G 14 * John M, Micheelis W, Biffar R. Einflussfaktoren mundgesundheitsbezogener Lebensqualität - Validierung einer deutschen Kurzversion des Oral Health Impact Profile (OHIP-G 14). Dtsch Zahnärztl Z 2004; 59: 328-333
